# Function of TRP channels in monocytes/macrophages

**DOI:** 10.3389/fimmu.2023.1187890

**Published:** 2023-06-19

**Authors:** Jiangbo Wu, Zhuo Li, Ya Deng, Xianmin Lu, Chen Luo, Xingyi Mu, Ting Zhang, Qi Liu, Siqi Tang, Jiajing Li, Qimin An, Dongdong Fan, Yiwei Xiang, Xianli Wu, Yanxia Hu, Qian Du, Jingyu Xu, Rui Xie

**Affiliations:** ^1^ Department of Gastroenterology, Digestive Disease Hospital, Affiliated Hospital of Zunyi Medical University, Zunyi, China; ^2^ The Collaborative Innovation Center of Tissue Damage Repair and Regeneration Medicine of Zunyi Medical University, Zunyi, China

**Keywords:** monocytes, macrophage, TRP channels, macrophage polarization, system disease

## Abstract

The transient receptor potential channel (TRP channel) family is a kind of non- specific cation channel widely distributed in various tissues and organs of the human body, including the respiratory system, cardiovascular system, immune system, etc. It has been reported that various TRP channels are expressed in mammalian macrophages. TRP channels may be involved in various signaling pathways in the development of various systemic diseases through changes in intracellular concentrations of cations such as calcium and magnesium. These TRP channels may also intermingle with macrophage activation signals to jointly regulate the occurrence and development of diseases. Here, we summarize recent findings on the expression and function of TRP channels in macrophages and discuss their role as modulators of macrophage activation and function. As research on TRP channels in health and disease progresses, it is anticipated that positive or negative modulators of TRP channels for treating specific diseases may be promising therapeutic options for the prevention and/or treatment of disease.

## Introduction

Macrophages are widely found in various tissues and organs of the human body and are also known as the mononuclear phagocyte system(MPS). Macrophages in the body come from a wide range of sources, including monocytes in the blood, hematopoietic stem cells in the bone marrow and early T lymphocytes in the thymus. The most important way is that monocytes in the blood penetrate the blood vessels and enter the connective tissue to differentiate and form macrophages (1). It is a heterogeneous and plastic population of immune cells with a variety of immune functions, such as bactericidal, phagocytosis, antigen presentation and cytokine secretion. It plays a key role in innate and acquired immunity.

TRP channels are ubiquitously expressed multimodal ion channels with six transmembranes (S1-S6) domains and intracellular N- and C-termini. Fragments between S5 and S6 are embedded to form ion passages, which are especially permeable to Ca2+ and Na+ (2). It can be activated by various physical and biochemical stimuli, including heat and cold, osmotic stimuli, matrix stiffness, phorbol derivatives, and growth factors. According to their function and amino acid sequence homology, they can be divided into seven subfamilies: TRPC (canonical), TRPV (vanilloid), TRPM (melastatin), TRPN (Nompc-like), TRPA (ankyrin), TRPP (polycystin), TRPML (mucolipin), except TRPN protein in Drosophila and zebrafish, other TRP proteins are present in mammals (3). At present, the TRP subfamily is widely distributed in human organs and tissues, and several of its members have been reported to be expressed in monocytes and macrophages (4, 5). It plays an important role in many systemic diseases by regulating ions involved in cell survival, apoptosis, phagocytosis and other functions (6). TRPs channels can be activated by various stimuli, including chemical agonists, temperature changes, mechanical stress, osmotic pressure, and G-protein-coupled receptor activation (7). TRPs channels can regulate various biological activities in human body, including sensory injury (8), chemical sensation (9), vascular tone and permeability (10), release of neuropeptides and immune cell mediators (11), skin barrier function (12), etc. Much has been written about the role of TRP channels in systemic diseases. However, the mechanisms involved in the mononuclear phagocyte system and its role in multi-system diseases are less well described. In the following, we discuss monocytes/macrophage TRP channels’ regulation and signaling pathways in multisystem diseases.

## The roles of TRP channels in macrophage polarization

**Figure 1:**
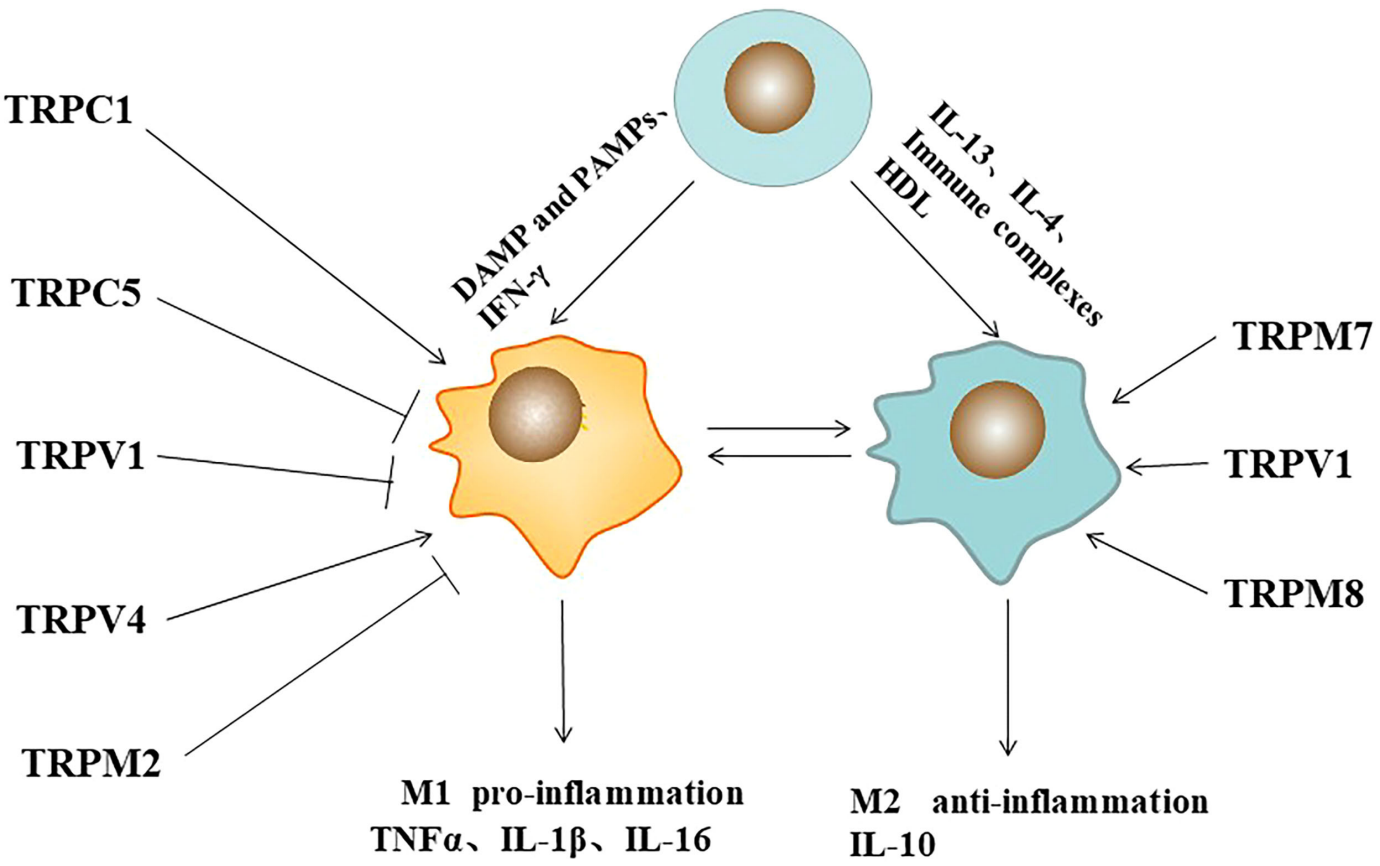
Pathogen-associated molecular patterns (PAMPs), immune complexes, cytokines, and TRP ion channels regulates macrophage polarization. Symbol (→) meaning stimulation, (—┤) meaning inhibition.

Mature macrophages undergo dynamic phenotypic/functional switching in response to microenvironmental signals, known as macrophage polarization. Macrophages are mainly activated into two phenotypes, M1 and M2, depending on their response to environmental stimuli (13). M1 macrophages can be induced by pathogen-associated molecular patterns or in combination with other cytokines (e.g., interferon γ (IFN-γ), tumor necrosis factor-α (TNF-α), granulocyte-macrophage colony-stimulating factor (GM-CSF)). M1-type macrophages are characterized by the secretion of large amounts of inflammatory cytokines (IL-1β, IL-6, IL-23, and TNF-α) and the enhancement of antigen presentation ability and host immune clearance (14). M2 macrophages can be induced by immune complexes, IL-13, IL-4. M2-type macrophages are characterized by the secretion of arginase Arg1, chitinase 3 (Chil3, also known as Ym1), resistin-like α(RELMα, also known as Fizz1), mannose receptor (MRC1, Also known as CD206) and the anti-inflammatory factor IL-10 (15). The polarized state functions of the two macrophages are very different and even antagonistic to each other. Different forms of macrophages play different functions under different physiological and pathological conditions. They can be transformed into each other under specific conditions and play an important role in regulating human immunity. It is closely related to the occurrence and development of infection, tumor, metabolism, immunity and other diseases (16). Over the past decade, efforts have been made to elucidate the molecular mechanisms by which macrophage TRP channels regulate polarization (**Figure 1**).

Up-regulation of TRPA1 in atherosclerotic plaques can regulate the development of macrophages to the M1 inflammatory phenotype, thereby promoting the progression of atherosclerosis (17). It has been reported that TRPC1-dependent STAT1/NF-κB pathway in macrophages can mediate M1 phenotype activation, whereas Orail1-mediated STAT6 activates the M2 phenotype. In TRPC1-/- mice, most M1-related genes were significantly reduced, and the M1 phenotype was suppressed, whereas most M2-related genes were significantly increased, but the M2 phenotype was not affected. Orai1-/- macrophages showed decreased anti-inflammatory cytokines and inhibited M2 polarization. Loss of TRPC3 function leads to increased apoptosis in naive macrophages, but it does not affect the expression of specific M1 or M2 phenotypic markers (18). Reduction of TRPC3-deficient M1 macrophages results in ER stress-induced apoptosis and impaired unfolded protein response (19). In RAW264.7 cell line, TRPC5 inhibition by ML204 or TRPC5 knockdown by shRNA significantly increased serum levels of inflammatory factors, TNF-α and IL-6. Similarly, in TRPC5 -/- mice, the number of M1 macrophage polarization marker CD68 was significantly increased in the infiltrating aortic wall, indicating that TRPC5 inhibited macrophage M1 polarization, which was significantly increased after knockdown. This process was further confirmed to be dependent on the Akt/IκB/NF- κB signaling pathway (20). In a rat model of osteoarthritis (OA), intraarticular injection of capsaicin (CPS) inhibited the infiltration of M1 macrophages. It reduced the release of inflammatory mediators IL-1β, IL-6, TNF-α and iNOS through the TRPV1/Ca2+/CaMKII/Nrf2 signaling pathway. In addition, RT-PCR, Western blotting and immunohistochemical analysis showed that iNOS expression was decreased and arginase-1 expression was increased in the nigra injury induced by capsaicin-treated LPS. Capsaicin may play a protective role in neurons by inhibiting the activation of M1 in injured nigra microglia/macrophages and promoting their conversion to M2 (21). In the lipopolysaccharide (LPS) -injured rat model of Parkinson’s disease (PD) inflammation, capsaicin promoted the conversion of the M1 microglia/macrophage population to the anti-inflammatory M2 state through TRPV1 (22). In the bleomycin-induced mouse skin fibrosis model, increased skin stiffness resulted in the upregulation of M1 markers, IL-1β and Mcp1, while TRPV4 knockdown reduced the markers in tissues (23). Upon H. pylori stimulation, TRPM2 deficient macrophages promote M1 polarization of classically activated macrophages in response to H. pylori (24).

TRP channels also regulate M2 polarization in macrophages. *In vitro*, LPS- induced TNF-α release was increased, but IL-10 release was attenuated in TRPM8 KO PM, suggesting that TRPM8 contributes to the expression of anti-inflammatory cytokines. *In vivo*, menthol (TRPM8 agonist) enema increased in the anti- inflammatory cytokine profile of M2 macrophages, which had a protective effect on mice with experimental colitis. TRPM8-deficient mice exhibit increased susceptibility to colitis, a phenotype rescued by systemic IL-10 expression (25). Functional expression of TRPM7 was found on macrophages. IL-4 stimulated TRPM7 activity in M2 macrophages was significantly increased. Macrophages exposed to IL-4 or M-CSF showed a 3.5-fold increase in elongation factor-CSF. However, macrophages treated with TRPM7 inhibitors NS8593 or FTY720 and IL-4 or M-CSF showed no morphological changes (26). Therefore, TRPs play important roles in macrophage polarization.

## TRPs participate in the regulation of macrophage activation pathway

As an important immune cell in human body, macrophages can be activated in a variety of ways to produce different signal transduction, so as to regulate the immune response of human body. TRP channels interact with macrophage activation signals to regulate the function of macrophages.

**Figure 2:**
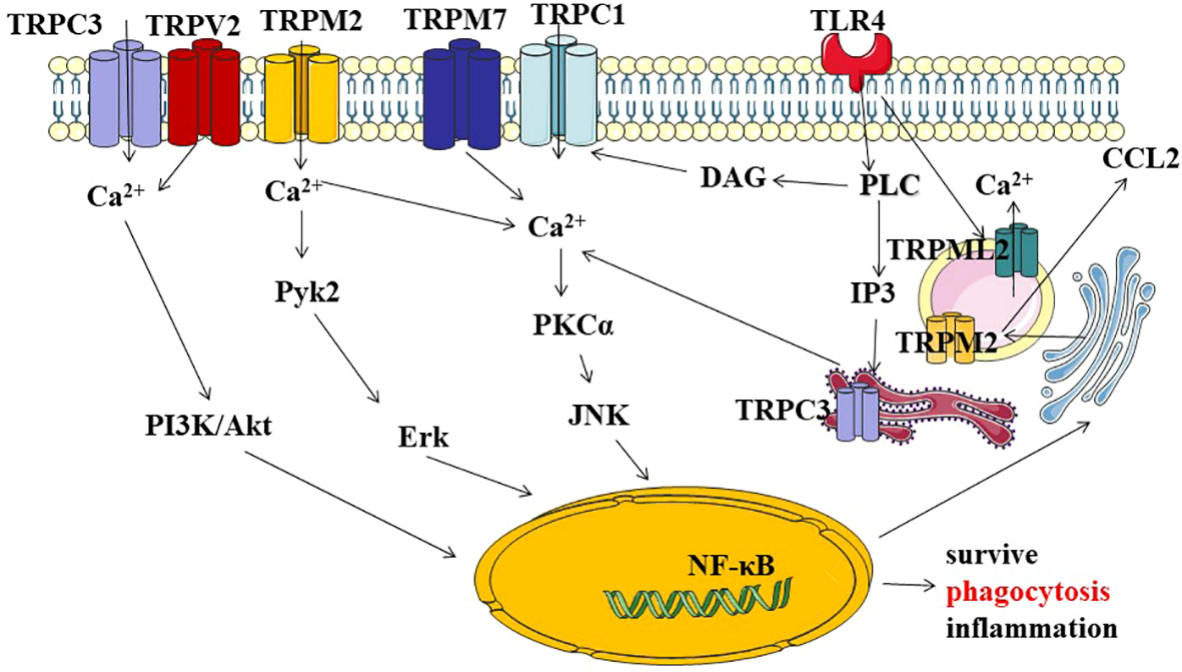
Describe TRP channels through different signaling pathways involved in macrophage survival,phagocytosis, and the regulation of inflammatory signals. (1) TRPs/Ca2+-mediated activation of PI3K/AKT axis pathway signal transduction, ultimately regulating genetic information expression, macrophage activity, and cell survival. (2) TRP channels crosstolk with Toll-like receptor 4 (TLR4) signaling regulates with inflammation through the Ca2+-dependent PKC-JNK-NF-kB signal. (3) TRP channels regulate inflammation through the Ca2+-dependent tyrosine kinase Pyk2/Erk signaling pathway. (4) intracellular TRPs/Ca2+ signaling pathway is involved in immune regulation. PI3K:Phosphatidylinositol-3-kinase; Akt, (PKB.Protein kinase B); Pyk2:Proline-rich tyrosine kinase 2; Erk:extracellular regulated protein kinases; PKC:Protein kinase C; JNK:c-Jun Nterminal kinase; NF-kB nuclear factor kappa-B; PLC:Phospholipase C; IP3, inositol 1, 4, 5-trisphosphate; DAG:diacylglycerol.

At present, there are many studies on TRP signal transduction in macrophages, mainly in the following aspects (**Figure 2**). Firstly, TRP/Ca2+ regulates macrophage survival and phagocytosis through PI3K/AKT signaling axis. Phosphatidyl inositol-3-kinase (PI3K)/AKT axis pathway is a recognized survival pathway and activation way in macrophages (27), and it is largely influenced by Ca2+ influx into the cell. Damage to survival signaling and secretion has been demonstrated in TRPC3-/- bone marrow-derived macrophages (28). TRPV2 is recruited to nascent phagosomes during early phagocytosis through pathways involving PI3K, Akt, and PKCζ signaling (29). Phagocytosis and motility defects observed in TRPV2 KO macrophages. Secondly, the TRP channel and Toll-like receptor 4 (TLR4) signaling pathway jointly regulate the inflammatory response. TLR4 is an important member of the Toll-like receptor family. It is a typical LPS receptor in Gram-negative bacteria. Recognition of LPS by TLR4 initiates a chain of events that culminates in the activation of transcription factors AP-1, NF-κB, or IRF-3 through different signaling branches (30). Ultimately, it leads to up-regulation of transcription of cytokines, enzymes and proteins, which contribute to inactivation of invading microorganisms. There have been several studies on the regulation of TRP channels on the TRL4 signaling pathway. In primary human monocytes, co-incubation with LPS resulted in the upregulation of TRPM2 mRNA, protein, and ADP nuclear-induced membrane currents. However, these results were reduced by the downregulation of TRPM2 expression in THP-1 monocytes using short hairpin RNA. TRPM2 Ca2+ influx plays a key role in LPS-induced cytokine production (31). TRPM2-/-mice reduced LM-induced cytokine production. Treatment with recombinant IFN-γ reversed TRPM2-/- susceptibility to high-dose Lm infection (32). Bacteria-mediated activation of TRPC1 is dependent on TLR4, which induces depletion of endoplasmic reticulum (ER) stores. After activating phospholipase Cγ (PLC-γ), TRPC1 mediates Ca2+ entry and triggers protein kinase Cα (PKCα) activity, which promotes nuclear translocation of NF- κB/Jun N-terminal protein kinase (JNK) and enhances inflammatory response (33). Thirdly, TRP channels regulate inflammation through the Ca2+-dependent tyrosine kinase Pyk2/Erk signaling pathway. TRPM2 is a Ca2+ -permeable, non-selective TRP channel, which is highly expressed in macrophages. It is controlled by adenosine diphosphate ribose (ADPR), circular ADPR (cADPR), and nicotinate adenine dinucleotide phosphate (NAADP). TRPM2 is also regulated by intracellular oxidative stress, such as H2O2 (34). In Ca2+ imaging, H2O2 enhanced the heat-evoked response in WT macrophages, and this current could be inhibited by 2-APB, suggesting a role for TRPM2 in this process. It was further confirmed that H2O2 reduces the temperature threshold for TRPM2 activation through Met oxidation and ADP-ribose production, allowing TRPM2 activation at physiological body temperature and contributing to increased release of G-CSF, CXCL2, IL-1α and possibly IL-1β (35). In the dextran sulfate sodium (DSS)-induced inflammation model, ROS-induced TRPM2/Ca2+ signaling promotes CXCL2, neutrophil infiltration and ulceration, through the ERK signaling pathway (36). Last, the intracellular TRPs/Ca2+ signaling pathway is involved in immune regulation.

It has been reported that TRP not only exists in the cell membrane but also in intracellular organelles (37). In the process of LPS activation, lipoprotein Lipin-1 stimulated the enrichment of endoplasmic reticulum DAG, the activation of endoplasmic reticulum TRPC3 channel, the release of ER calcium, the increase of Ca2+, the activation of NF-κB, and the upregulation of cytokines (38). Phagocytosis of immune cell function is closely related to TRPML channels (39). TRPML1 is a key lysosomal Ca2+ channel that regulates focal exocytosis and phagosome biogenesis. Phagocytosis of large particles activates the phosphoinositol and Ca2+ -dependent exocytosis pathways to provide membranes required for pseudopodia extension to clear senescent and apoptotic cells *in vivo (* 40). TRPML1 shRNA in RAW264.7 cells did not lead to excessive acidification of late endosomal/lysosomal compartments. Reduced TRPML1 levels result in delayed transport of endocytic proteins to lysosomes and expansion of compartments of multivesicles and multilayers (41). Endogenous TRPML2 expression has been reported in early/circulating endosomes. After TLR4 activation, intracellular TRPML2 levels were strongly up-regulated. TRPML2/Ca2+ signaling is directly associated with CCL2 trafficking and secretion (42, 43). Patch-clamp results showed luminal localization of TRPM2 terminals in the phagosome. TRPML3 is distributed in the early compartments (early and late endosomes) of the endocytic pathway, and inhibition of TRPML3 function leads to increased calcium accumulation in the endosomal lumen, impaired endosomal acidification, and abnormal endosomal fusion (44). TRPM2 recruitment is directly coupled to phagosome maturation (45, 46).

Taken together, these findings suggest that TRPs are not only expressed on cell membrane and organelles in macrophage, but also regulate its polarization through different signaling pathways to influence cell functional properties. These signaling pathways and functional state of macrophages could be modulated to achieve the purpose of disease prevention and treatment.

## TRP in diseases

**Figure 3:**
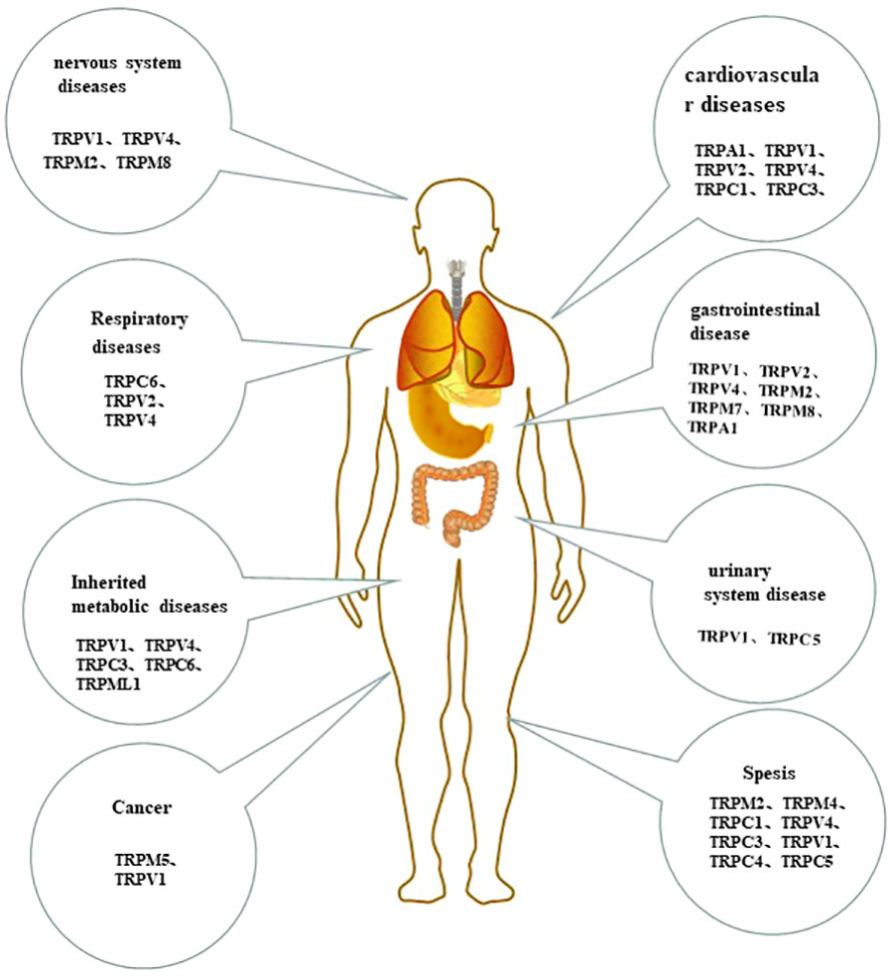
The tissue-distribution of monocytes/macrophages TRP channels and their roles in human system disease.

In the past decade, numerous studies have shown that abnormal expression and function of TRP are closely related to a variety of systemic diseases (**Figure 3**).

## Monocyte/Macrophage TRP channels in cardiovascular diseases

Atherosclerosis (AS) is the pathological basis of various cardiovascular diseases, characterized by high morbidity, disability, and high mortality. It is a major cause of

health and death, with an increasing trend in morbidity and mortality in recent years (47). As immune cells, macrophages play an important role in atherosclerosis. It takes up much cholesterol, turns it into fat, and turns itself into foam cells. Foam cells are the primary cells in atherosclerotic lesions. Macrophage blistering is a key step in the formation of atherosclerosis and is also a research hotspot. New research has shown that many channels responsible for cholesterol metabolism have regulatory roles in macrophages, thereby influencing foam cell formation. Lipid accumulation is also associated with various inflammatory cells and is necessary for developing AS. In addition, apoptosis of macrophages is the main cellular component causing instability of AS plaques. Some studies have found that the results of macrophage apoptosis in early and late AS are different. Macrophage apoptosis in the early stage of AS lesions can reduce the lesion area, while macrophage apoptosis in the late stage can promote cardiovascular diseases (48, 49). It has been found that cells regulated by a variety of classical TRP channels can participate in the pathological process of AS.

TRPA1 channel expression in the normal and atherosclerotic aorta. TRPA1 can regulate the development of macrophages to M1 inflammatory phenotype, thereby promoting the progression of atherosclerosis (17). TRPA1 deficiency in bone marrow-derived macrophages (BMDMs) would significantly increase the serum levels of cytokines. In apoE -/- mice, immunohistochemical staining showed that TRPA1 channel positive signals were mainly confined to macrophage regions in apoE -/- mice lesions. Allyl isothiocyanate (AITC), a TRPA1 agonist, can reduce atherosclerotic areas and inflammatory factors. In BMDMs, TRPA1/Ca2+ signaling may be associated with increased cholesterol efflux by down-regulating ATP-binding cassette transporters, which ultimately inhibits macrophage foaming (50). The phagocytic uptake of oxidized low-density lipoprotein (oxLDL) is a key process in atherogenesis. Immunohistochemical staining for TRPV1 demonstrated positive signals confined mainly to macrophages. oxLDL activates TRPV1 in BMDMs and initiates lipid accumulation induced by Ca2+ influx (51). These results suggest that TRPV1 plays a key role in forming foam cells.

The activation of TRPV4 also intensifies the development of AS. TRPV4 channels function in primary normal BMDMs, and in RAW264.7 cells. As a mechanical sensor, TRPV4 can regulate macrophage phagocytosis through matrix stiffness, among which oxLDL phagocytosis uptake is a key process in atherogenesis. Knockout of TRPV4 prevents foam cell formation induced by oxLDL uptake after stromal stiffness or scratching (52). In addition, studies have shown that 7- keto cholesterol is a component of oxidized LDL. THP-1 treatment induces TRPC1 easily migrate from non-raft to lipid raft domain (LRD), which leads to a continuous increase of intracellular Ca2+ due to channel opening and then calcination phosphatase activation, BAD dephosphorylation, and apoptosis.

TRPC1 is also associated with the adhesion of coronary endothelium and its subsequent extravasation in the early stages of atherosclerotic progression (6). Dube PR et al. reported that TRPC3 is related to advanced atherosclerosis. Selective deletion of TRPC3 in macrophages favors plaque regression and impairs the activity of a novel macrophage-associated, BMP-2-dependent mechanism of calcification. TRPC3- deficient, non-polarized bone marrow-derived macrophages (BMDMs) exhibited, *in vitro*, impaired survival signaling and increased apoptosis (53), whereas TRPC3-deficient M1 macrophages exhibited reduced susceptibility to atherorelevant pro-apoptotic stimuli (54). It indicated that macrophage TRPC3 had different effects on atherosclerosis under different environmental stimuli. Taken together, TRP channels are promising potential therapeutic targets for AS.

Macrophage TRP channels has also been reported to play a role in other cardiovascular diseases. TRPC3 channel expression and TRPC3-related store-operated calcium influx increased in monocytes from spontaneously hypertensive rats (55, 56). TRPV2 is upregulated in the myocardial infarction area, which may be involved in the migration ability of macrophages to hypoxic cardiomyocytes (57). While TRPV2-KO mice had better cardiac function than WT, intravenous administration of WT macrophages in the peritoneal cavity significantly reduced the survival of TRPV2-KO mice after MI in a mouse model of myocardial infarction (58). Of note, modulation of macrophage TRPV2 channels may prevent and treat myocardial infarction.

## Monocyte/Macrophage TRP channels regulate gastrointestinal disease

In recent years, many studies have found a certain relationship between TRP channels and gastric ulcer, functional dyspepsia, irritable bowel syndrome and other digestive system diseases. The present study showed that TRPV2 was also detected on resident macrophages in the mucosa of normal and reflux esophagitis rats. TRPV2 of macrophages may be associated with the progression of reflux esophagitis (59). CX3CR1hi macrophages are abundant in the small intestine’s lamina propria (siLP) and highly express cannabinoid/vanilloid receptors. CP acts by binding to the vanilloid receptor TRPV1, leading to the local production of AEA, and acts through CB2, which plays an important role in maintaining the intestinal immune balance (60). Stimulation of intestinal muscularis macrophages TRPV4 promotes the release of prostaglandin E2. It causes colonic contractions in a paracrine fashion through prostaglandin E receptor signaling in intestinal smooth muscle cells, playing an important role in maintaining normal gastrointestinal motility (61). In Crohn’s disease(UC), the TRPV4 expression was higher in peripheral blood mononuclear cells and is related to the disease (62). The types of TRP channels are often different in different tissue sites, and the same TRP channels may play different roles in different sites. In chronic infection with H. pylori, TRPM2-/- triggers macrophage production of more inflammatory mediators and promotes macrophage M1 polarization in response to H. pylori, showing increased gastric inflammation and reduced bacterial colonization (24). In the TRPM2 -/- mouse model of DSS, the expression of CXCL2 was strongly inhibited, suggesting that TRPM2 -/- mice largely mediate protection from DSS colitis (36). Matsumoto K and others reported that TRPM2 mediates the development of postoperative intestinal obstruction. Intestinal manipulation triggers the release of inflammatory cytokines and chemokines through TRPM2 activation of muscle- resident macrophages, which in turn promotes the infiltration of macrophages and neutrophils into the muscle layer, ultimately leading to dyskinesia (63). TRPM7-mediated Ca2+ signaling, which is critical for LPS-induced macrophage activation. Selective deletion of TRPM7 in bone marrow macrophages renders mice resistant to LPS- induced peritonitis (64). TRPM8 in macrophage activation can increase nonselective cation current to depolarize the membrane in the cell (65). TRPM8-/- activity in PMs resulted in impaired proinflammatory cytokine profile and phagocytosis. Whereas TRPM8 activation induced the opposite effect *in vivo*, activating TRPM8 by repeated menthol enemas protects mice from experimental colitis (25). The non-psychoactive cannabis constituent Cannabichromeene reduces NO, IL-10, and INFγ levels in LPS- activated peritoneal macrophages depending on TRPA1 activation (66).TRPA1 is upregulated in colitis, and its activation exerts a protective effect by reducing the expression of cytokines IL-1β and chemokine MCP-1 (67). Altogether, TRP channels exist in normal gastrointestinal macrophages, and the changes in their expression and function on macrophages play crucial roles in the development of gastrointestinal disease.

## Monocyte/Macrophage TRP channels in respiratory diseases

Macrophage TRP has been reported to be associated with COPD, ventilator- induced lung injury (VILI), et al. The expression of TRPC6 mRNA was significantly increased in alveolar macrophages from COPD patients, and the patch clamp showed that TRPC6 was functionally active on smaller macrophages (68). In pulmonary cystic fibrosis (CF), alveolar macrophage TRPC6 is associated with proton pump generation of transmembrane potential, thereby restoring phagocytic and bactericidal functions of damaged alveolar macrophages in CF (69). In addition, studies have shown that the expression and function of TRPV2 in CF are defective, showing an imbalance of calcium homeostasis, ultimately leading to impaired phagocytosis (70). SARS-CoV-2 infection of alveolar macrophages may be the driver of a “cytokine storm” that may lead to lung tissue, heart, and lung damage (71). Recent studies have identified the role of macrophage TRPV2 in SARS-CoV-2 infection. Under febrile conditions, SARS-CoV-

2 infection of PBAM promotes Ca2+ influx, further activating the NF-κB p65 signaling pathway and enhancing cytokine secretion. We can reduce the inflammatory response in the lung by reducing the release of cytokines that drive the inflammatory response by knockdown or antagonist of TRPV2 (72). The literature has reported that activation of TRPV4 on lung-running macrophages can induce the production of inflammatory mediators, thereby aggravating lung injury (73).

The role of TRPV4 in lung tissue is controversial.TRPV4 significantly increases calcium, superoxide, and nitric oxide production in response to high PIP activation, with increased filtration coefficient K(f), ultimately leading to acute lung injury (74, 75). It is also reported in the literature that it has a protective effect in lung injury. Scheraga RG and others reported that changes in outer matrix stiffness trigger TRPV4 to regulate inflammatory mediators through Ca2+ signaling, decrease IL-1β, increase IL-10, and ultimately the clearance of bacteria and resolution of infection-associated lung injury (76). This process is mainly through MAPK/DUSP1/JNK signal to regulate (77). TRPV4 also reduces acute lung injury caused by reactive chemicals, including acids, chlorine, et al. In the HCl- induced lung injury model, GSK2337429A reduces the release of proinflammatory factors, such as KC (CXCL1), GCSF, and VEGF, improves airway mechanics, and oxygen saturation, and reduces protein leakage (78). TRPV4 inhibition prevents LPS- induced lung injury by calcineurin-NFATc3 pathway (79). Naik SK et al., 2020 report a bifunctional role of the TRPV4 channel in response to M. tuberculosis infection in mice. Early TRPV4 regulates intracellular pathogens by regulating phagosome acidification and maturation, and late TRPV4 promotes neutrophil responses to proinflammatory stimuli, reactive oxygen species production, adhesion, and chemotaxis (80).The TRP channels modulated Ca^2+^ signaling pathway could be potential therapeutic targets in respiratory disease.

## Monocyte/Macrophage TRP channels in inherited metabolic diseases

Persistent, low-grade inflammation may be a potentially modifiable risk factor in inherited metabolic diseases such as diabetes and obesity (81). Obesity is closely related to chronic inflammation in adipose tissue, in which increased recruitment of macrophages can cause the release of various inflammatory mediators. Oral administration of AEA provided significant protection against T1D in a mouse model. AEA-mediated Mφ regulation of pancreatic lymph node (PLN) requires the presence of functional TRPV1. TRPV1 activation leads to the significant increase of CD11b+ CX3CR1+ cells and ultimately leads to the increase of IL-10, which has anti- inflammatory and tissue-protective effects (60). Studies have shown that TRPV4-/- mice reduce adipose tissue release of inflammatory mediators and improve insulin sensitivity (82). In diabetic patients, oxidative stress induced by high glucose can increase the expression of TRPC3 and TRPC6 and regulate the release of inflammatory cytokine TNF-α through calcium signaling, which is closely related to cardiovascular diseases caused by high glucose (83). Hyperglycemia can also induce the overproduction of reactive oxygen species (ROS). The TRPM2/Ca2+ signal induction is essential for activating NLRP3 inflammasome, increasing the secretion of IL-1β, thereby inducing diabetic complications (84). In Niemann-pick Type C (NPC) macrophages, TRPML1 expression/activity is sufficient to mediate lysosomal Ca2+ signaling, thereby correcting transport defects and reducing lysosomal storage and cholesterol accumulation (85). Metabolic disease is a complicated and refractory disease with many factors. Although the above-mentioned macrophage TRP channels are implicated, the mechanisms of action have not been elucidated in detail yet.

## Monocyte/Macrophage TRP channels in nervous system disease

TRP channels play an important role in mediating crosstalk between the nervous and immune systems, especially in inflammation. In the nervous system, two-way interaction between neurons and immune cells, especially in microglia, plays an important role in nerve inflammation. Studies have shown that the role of TRPV1 in the nervous system and immune response is complex. Capsaicin, as a TRPV1 agonist, induces Ca^2+^ signaling in microglia mitochondria, production of mtROS, activation of MAPK, and increased chemotaxis activity in microglia, inducing immune and neurological diseases (86). In addition, the absence of TRPV1 channels can reduce the activation of macrophages and glial cells induced by post-traumatic brain injury (TBI) of the trigeminal nervous system, and ultimately reduce post-traumatic headache (PTH) (87). These suggest that TRPV1 is harmful in neurological diseases. However, other studies have shown that capsaicin can also regulate the M1/M2 polarization of macrophages and inhibit the expression of LPS-induced pro-inflammatory mediators in the substantia nigra, thus preventing the degeneration of substantia nigra dopamine neurons and playing a protective role in Parkinson’s disease (22, 87). There are endogenous and functional heat-sensitive ion channels TRPV4 and cold-sensitive ion channels TRPM8 in primary rat microglia. Pharmacological regulation of these two channels affects intracellular Ca2+ levels, cell morphology, migration, and motility. Thus, TRPV4 and TRPM8 act as potential regulators of microglial activity. Interactions of glial cells in neurodevelopment and neurodegeneration (88). Injection of LPS into mouse ventricles stimulated microglial activation. It significantly increased the release of TNF-α and the expression of galectin-3, which were inhibited by the selective TRPV4 agonist 4α-phorbol 12, 13- didecanoate (4α-PDD) (89). The TRPM2 channel, which is abundantly expressed in infiltrating macrophages of the central nervous system (CNS), plays a crucial role in the development of experimental autoimmune encephalomyelitis (EAE), an animal model of multiple sclerosis (MS). EAE progression was inhibited by knockout or pharmacological inhibition of TRPM2. This was attributed to decreased CXCL2 chemokine production by CNS-infiltrating macrophages in TRPM2-KO mice, resulting in inhibition of neutrophil infiltration into the CNS (90). These results reveal an important role for TRPM2 in the pathogenesis of EAE and clarify its potential as a therapeutic target.

## Monocyte/Macrophage TRP channels in urinary system disease

Monocyte/macrophage TRP channels play a role in the regulation of urinary diseases. TRPV1 channels serve as a potential regulator of monocyte/macrophage recruitment in hypertensive renal injury. By attenuating MCP-1/CCR2 signal- dependent inflammatory responses, kidney damage in patients with salt-sensitive hypertension is mitigated (91). In patients with chronic renal failure, erythropoietin(EPO)-treated hemodialysis patients showed significantly increased amounts of TRPC5 mRNA in monocytes compared with EPO-free hemodialysis patients (92). Therefore, modulation of macrophage TRP channels could affect inflammation leading to kidney damage.

## Monocyte/Macrophage TRP channels in tumor

Tumor immune infiltrating cells have prognostic value in the occurrence and progression of tumors and are associated with improved clinical outcomes. Macrophages account for a large proportion of immune cells in triple-negative breast cancer (TNBC), and TRP-related genes are closely related to the infiltration of immune cells. TRPM5 was differentially expressed in different samples, its related gene ADCY6 was negatively correlated with macrophage M1, HRH1 was positively correlated with macrophage M2, and HTR2B was positively correlated with macrophage M2 (93). These results suggest that macrophage TRP channels play a role in tumor progression. TRPV1 may be involved in the occurrence of cervical squamous cell carcinoma by regulating macrophage infiltration and tumor immune escape (94).Currently, tumor immune escape is a hot topic in the field of tumor. It is known that persistent chronic inflammation may results in tumors. Mononuclear macrophages, as important immune cells, play an important role in tumorigenesis. In addition, immune escape is also important in the development of tumors. Therefore, it is necessary to study monocyte/macrophage TRP channels in cancer, which can provide new targets for the prevention and treatment of cancer.

## Monocyte/Macrophage TRP channels in sepsis

Sepsis refers to systemic and injurious inflammatory response syndrome caused by severe infection, a common critical disease in clinical practice. The key to sepsis is the imbalance of proinflammatory/anti-inflammatory mechanisms: in the early stage, systemic inflammation is predominant, manifested as SIRS; Later, severe immunosuppression predominates, namely CARS, leading to severe systemic infections and organ damage that are difficult to control (64, 95, 96). As an important part of innate immunity, Macrophages play an important role in an inflammatory response, and TRPs channels, as regulators of the inflammatory response, have been confirmed in several studies.

Qian X et al. demonstrated increased mortality in TRPM2-/- mice after cecal ligation and puncture. This correlates with TRPM2-mediated Ca2+ influx regulating heme oxygenase 1 (HO-1) expression on macrophages (97). Decreased Ca2+ in TRPM2-/- peritoneal macrophages affects phagosome maturation, increasing bacterial burden and mortality during E. coli sepsis (46). TRPM2 can also be activated on astrocytes during sepsis, reducing the release of inflammatory mediators through calcium signaling. It plays a protective role in sepsis-related encephalopathy (98). The protective effects of TRPM4 have been reported in the literature. In a model of cecal ligation and puncture induced sepsis, the lack of TRPM4 channels affected the peritoneal macrophage population and increased systemic levels of Ly6C(+) monocytes and proinflammatory cytokine production. Impaired Ca2+ mobilization in TRPM4(-/-) macrophages downregulates AKT signaling and subsequent phagocytic activity, resulting in bacterial overgrowth and translocation into the bloodstream (99). The result was a significant increase in mouse mortality. After LPS injection, TRPC-/- mice displayed higher IL-1β in the serum, a process independent of caspase-1 activation, which promoted IL-1β release by degrading TRPC1 independently of caspase-1 (100). In THP-1, Lipin-1-derived DAGs activate TRPC3 on the inner membrane of ER cells during LPS activation, and blockade of TRPC3 activity ameliorated the development of LPS-driven inflammation and sepsis in animal models (38). Compared with WT, TRPV1-/- mice exhibited significantly enhanced LPS-induced TNF-α, IL-6 and IL-10 and decreased MCP-1 and MIP-1β. It also increased apoptosis and decreased *in vitro* phagocytosis of LPS-treated peritoneal macrophages (101). This impaired macrophage function promotes the progression of sepsis. Functional TRPV4 is expressed in murine BMDMs and alveolar macrophages. LPS-stimulated cytokine release is dependent on TRPV4. TRPV4 -/- reduces IL-1 release and increases IL-10 (76). Both of these effects were dependent.Escherichia coli thioredoxin can induce death in LPS injected mice, accompanied by decreased leukocyte accumulation, regulation of cytokine release into the peritoneum and phagocytosis mediated by peritoneal macrophages. This process was confirmed to be mediated by forming functional complexes between TRPC5 and TRPC4. Dual blocking of TRPC4/TRPC5 by ML204 increased mortality and hypothermia but preserved macrophage phagocytosis in thioredoxin-treated LPS mice. In LPS mice treated with thioredoxin, TRPC5 depletion did not alter body temperature but promoted additional accumulation of peritoneal leukocytes and release of inflammatory mediators. These results suggest that TRPC4/TRPC5 channels play a protective role in sepsis (102).

## Conclusions and perspectives

This review highlights the possible functions of macrophage TRP channels in different systems, including the regulation of macrophage activation and their role in disease. In general, it plays an important role in physiological and pathological states. Due to the wide distribution of macrophages and the differential expression of TRP in different organs and tissues, targeting and selective activation and inhibition of macrophage TRP is the key to preventing and treating diseases. Currently, many drugs use TRP channels as therapeutic targets to treat diseases (103), for example, Cannabichromene, which treats colitis through TRPA1. The TRPC3-Nox2 complex may modulate cardiac events caused by various environmental risk factors. Ibudilast, a PDE4 inhibitor, strongly inhibits doxorubicin-induced ROS production and cytotoxicity by destabilizing the TRPC3-Nox2 complex (104). In the future, we look forward to more clinical studies to clarify the specific role of macrophage TRP channels in more diseases.

## Author contributions

JW and ZL wrote the manuscript. YD, XL, CL, XM, TZ, ST, JL, QA, DF, YX, XW, YH, and QD collect the literature. JX primarily revised and finalized manuscript. RX revised the manuscript for clarity and style. All authors contributed to the article and approved the submitted version.
